# Supplementary material for: Enzyme-inspired single-atom photocatalysis for oxygen reduction to hydrogen peroxide
Source: Nat Commun. 2025 Dec 7;16:10949. doi: 10.1038/s41467-025-67189-3 (PMC12686429; doi:10.1038/s41467-025-67189-3)
Supplement: Supplementary file 1 — Supplementary Information [file 41467_2025_67189_MOESM1_ESM.pdf]

Supplementary information

Enzyme-Inspired Single-Atom Photocatalysis for Oxygen Reduction to Hydrogen Peroxide

Lukáš Zdražil,[1,2,3] *Alejandro Cadranel,**[1,4,5] *Giorgio Zoppellaro,*[2,3] *Ayse Günay-Gürer,*[1] *Zdeněk Baďura,*[2,3] *David Panáček,*[2,3,6] *Hana Kmentová,*[2,3] *Camila Otero,*[7] *Štěpán Kment,*[2,3] *Maria Ana Huergo,*[7] *Emiliano Fonda,*[8] *Radek Zbořil,**[2,3] *and Dirk M. Guldi,**[1]

[1]Department of Chemistry and Pharmacy & Interdisciplinary Center for Molecular Materials (ICMM), Physical Chemistry I, Friedrich-Alexander-Universität Erlangen-Nürnberg, Egerlandstraße 3, 91058 Erlangen, Germany.

[2]Nanotechnology Centre, Centre for Energy and Environmental Technologies, VSB – Technical University of Ostrava, 17. listopadu 2172/15, 708 00 Ostrava-Poruba, Czech Republic.

[3]Regional Center of Advanced Technologies and Materials, The Czech Advanced Technology and Research Institute (CATRIN), Palacký University Olomouc, Šlechtitelů 27, Olomouc 779 00, Czech Republic.

[4]Universidad de Buenos Aires, Facultad de Ciencias Exactas y Naturales, Departamento de Química Inorgánica, Analítica y Química Física, Pabellón 2, Ciudad Universitaria, C1428EHA, Buenos Aires, Argentina.

[5]CONICET − Universidad de Buenos Aires, Instituto de Química-Física de Materiales, Medio Ambiente y Energía (INQUIMAE), Pabellón 2, Ciudad Universitaria, C1428EHA, Buenos Aires, Argentina.

[6]Center for Advanced Technologies and Engineering (CATEN), Technologická 375/3, 708 00, Ostrava-Pustkovec, Czech Republic, www.caten.tech

[7]Instituto de Investigaciones Fisicoquímicas Teóricas y Aplicadas (INIFTA), Universidad Nacional de La Plata, CONICET, Sucursal 4 Casilla de Correo 16, 1900 La Plata, Argentina

[8]Synchrotron SOLEIL, Départementale 128, Saint-Aubin, 91190, France

*Correspondence: ale.cadranel@fau.de, radek.zboril@upol.cz, dirk.guldi@fau.de

Details on LEPR computer simulation:

CuP@CD LEPR spectrum (Figure 3g):

Component 1: CDs radical

g_z = 2.001, g_y = 2.000, g_x = 2.000, hyperfine components: 3 ^{1}H, $A_{x,y,z}$ = 5.0 G, 5.0 G, 10.0 G, Lorentzian/Gaussian line = 0.8, Line-width tensor ($\mathbf{L}_{x,y,z}$) = 6.0 G, 8.0 G, 6.0 G.

Component 2: (with porphyrinoid contribution due to effective spin polarization)

g_z = 2.004, g_y = 2.000, g_x = 2.000, hyperfine components: 4 ^{14}N, $A_{x,y,z}$ = 1.0 G, 4.0 G, 8.0 G, Lorentzian/Gaussian line = 0.8, Line-width tensor ($\mathbf{L}_{x,y,z}$) = 7.0 G, 6.0 G, 6.0 G.

Contribution (Sum) components: 1, 2 = 50% (1) and 50% (2)

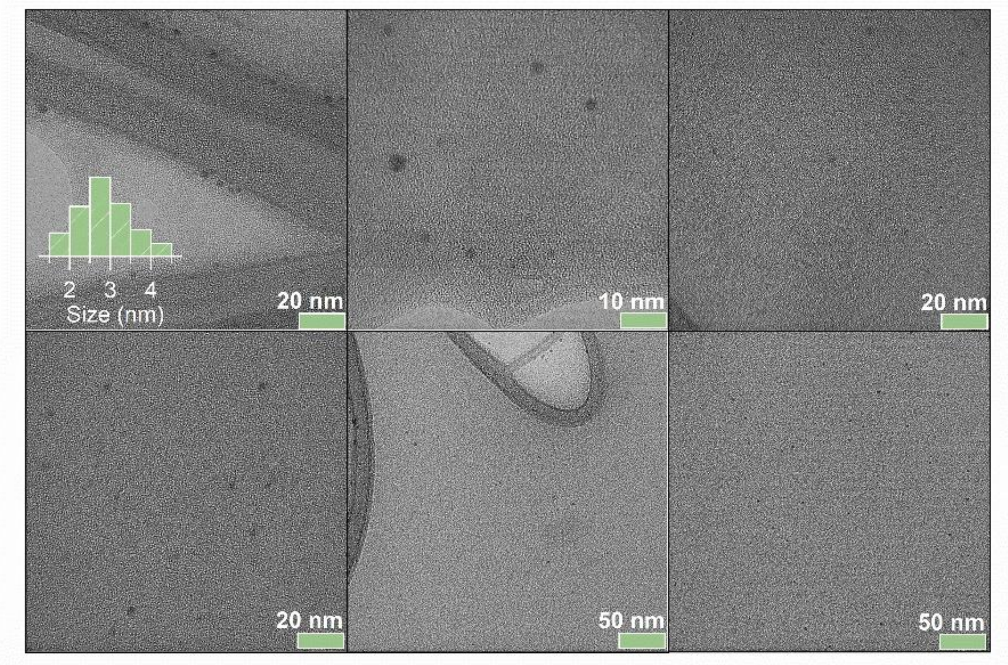

Figure S1. Morphology and size distribution of H_2P@CD. TEM images of H_2P@CD together with corresponding size histogram shown in the inset.

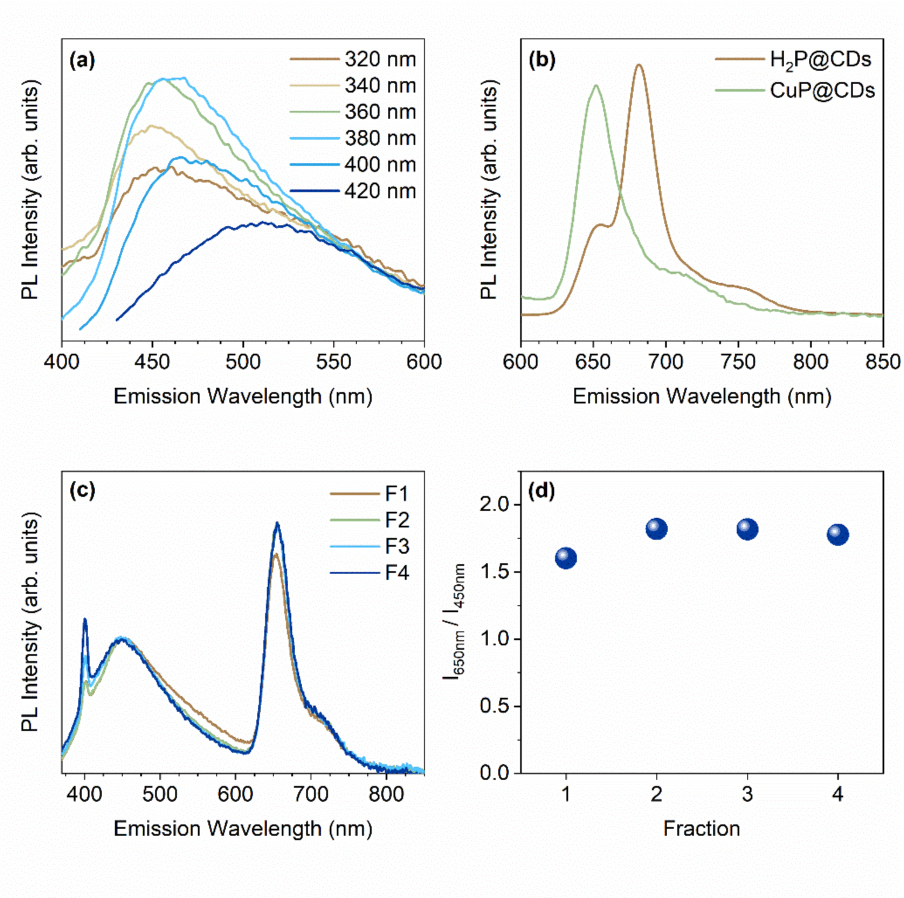

Figure S2. Photoluminescence characteristics and fraction analysis of H_2P@CD and CuP@CD. **(a)** Emission spectra of H_2P@CD under different photo-excitation wavelengths. **(b)** Emission spectra (λ_{ex} = 420 nm) comparing H_2P@CD and CuP@CD. **(c)** Emission spectra of F1-F4 fractions of CuP@CD collected after column chromatography (λ_{ex} = 355 nm). **(d)** Ratio of integrated PL intensities at 650 and 450 nm across fractions. All measurements were performed in water.

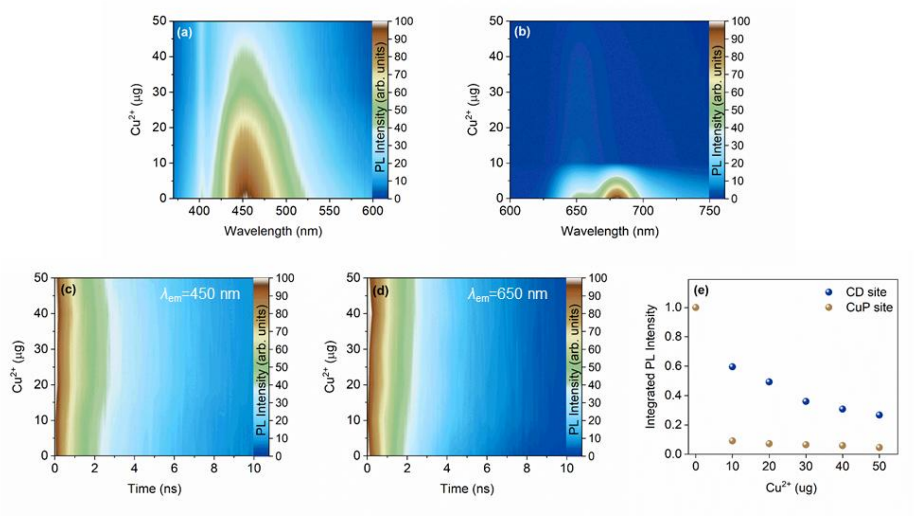

Figure S3. Influence of Cu^{2+} concentration on the optical response of H_2P@CD. (a–b) Emission color maps of H_2P@CD in aqueous solutions with varying Cu^{2+} concentrations. **(c–d)** Normalized time-resolved fluorescence intensity (λ_{ex} = 372 nm) at the emission maxima of carbon domains (λ_{em} = 450 nm) and porphyrinoid centers (λ_{em} = 650 nm) as a function of Cu^{2+} concentration (pH 9). **(e)** Integrated fluorescence intensity as a function of Cu^{2+} concentration for both CD and CuP sites, respectively.

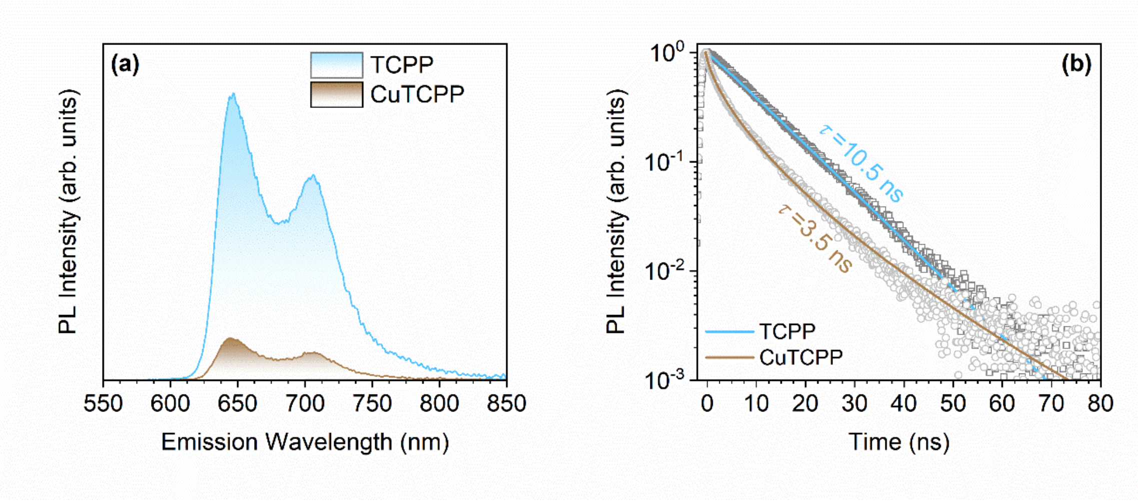

Figure S4. Comparison of fluorescence properties between TCPP and the reference CuTCPP. Steady-state and time-resolved fluorescence spectroscopy of TCPP (light blue) and CuTCPP (brown) in water. **(a)** Fluorescence spectra of TCPP and CuTCPP. **(b)** Corresponding decay curves for TCPP and CuTCPP collected at their maxima ($\lambda_{ex} = 372$ nm).

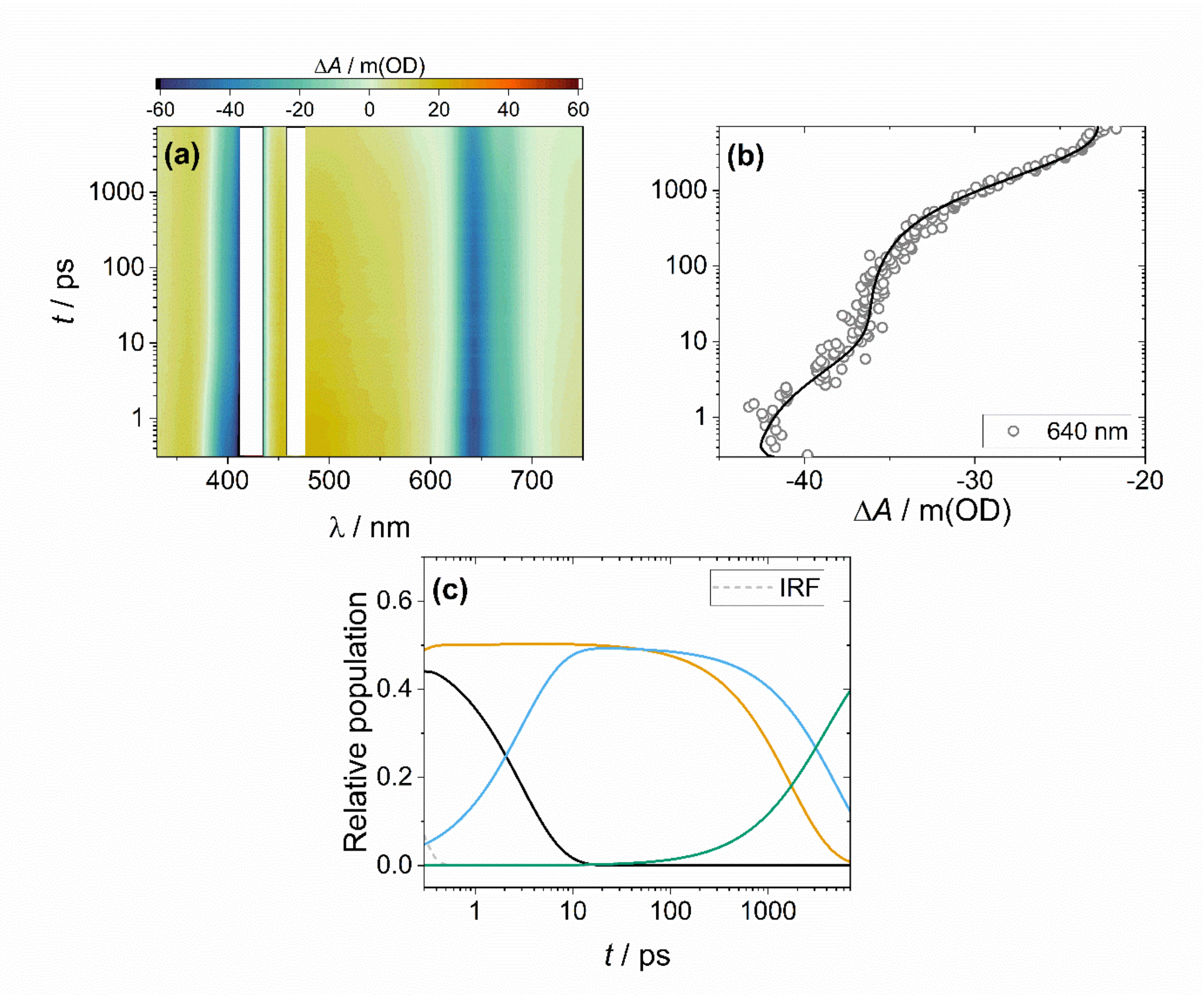

Figure S5. Femtosecond transient absorption spectroscopy of H_2P@CD in water. **(a)** Color map of the evolution of differential absorption spectra upon 420 nm photo-excitation with time delays between 150 fs and 5500 ps. **(b)** Experimental kinetic trace at 640 nm (open circles) and fitting (solid line). **(c)** Concentration evolution over time – hotS_{680} in black, S_{680} in orange, T_{680}/T_{650} in green, S_{650} in blue – see Figure S7 for details.

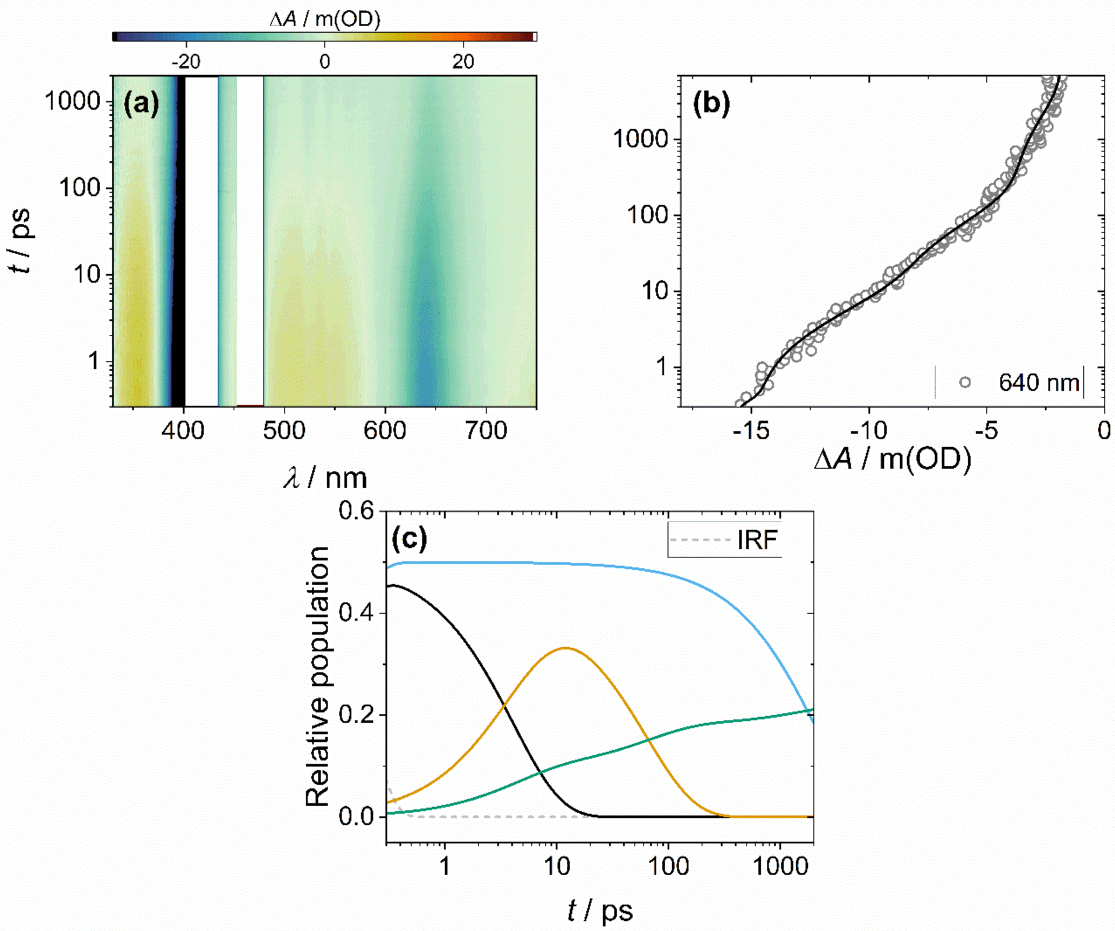

Figure S6. Femtosecond transient absorption spectroscopy of CuP@CD in water. (a) Color map of the evolution of differential absorption spectra upon 420 nm photo-excitation with time delays between 150 fs and 5500 ps. **(b)** Experimental kinetic trace at 640 nm (open circles) and fitting (solid line). **(c)** Concentration evolution over time – hotS_{680} in black, S_{680} in orange, T_{680}/T_{650} in green, S_{650} in blue – see Figure S7 for details.

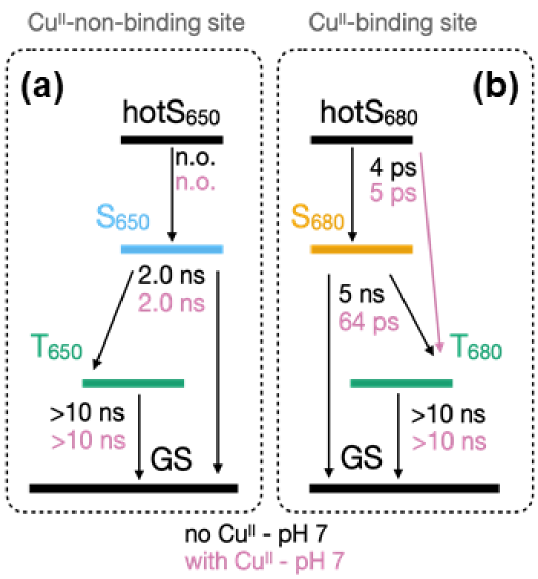

Figure S7. Jablonski diagram illustrating photoexcited-state relaxation in H_2P@CD and CuP@CD. Energy-level structure and relaxation pathways of H_2P@CD (lifetimes in black) and CuP@CD (lifetimes in pink), showing the effect of Cu^{2+} coordination on excited-state lifetimes within prophyrinic sites that do not bind (P_{650}, a) or bind (P_{680}, b) Cu(II) ions. The main impact is on intersystem crossing rates.

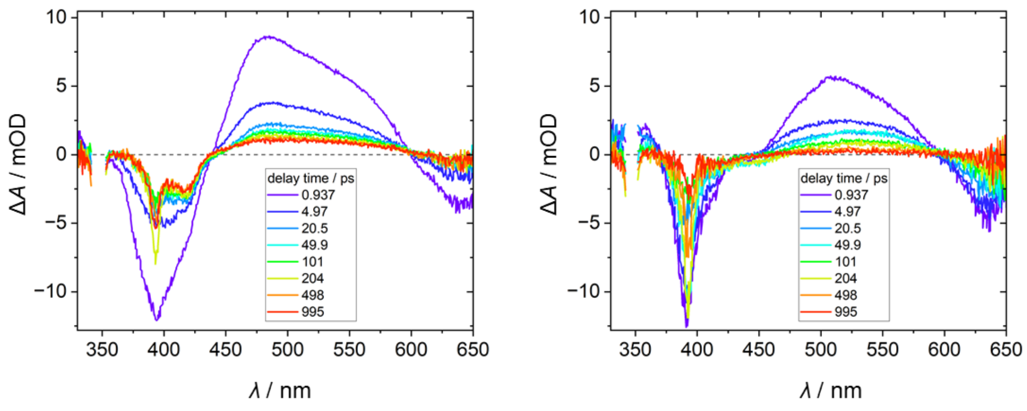

Figure S8. Femtosecond transient absorption spectroscopy of H_2P@CD and CuP@CD in water. Differential absorption spectra of H_2P@CD **(left)** and CuP@CD **(right)** obtained from fsTAS upon 350 nm excitation in water at room temperature.

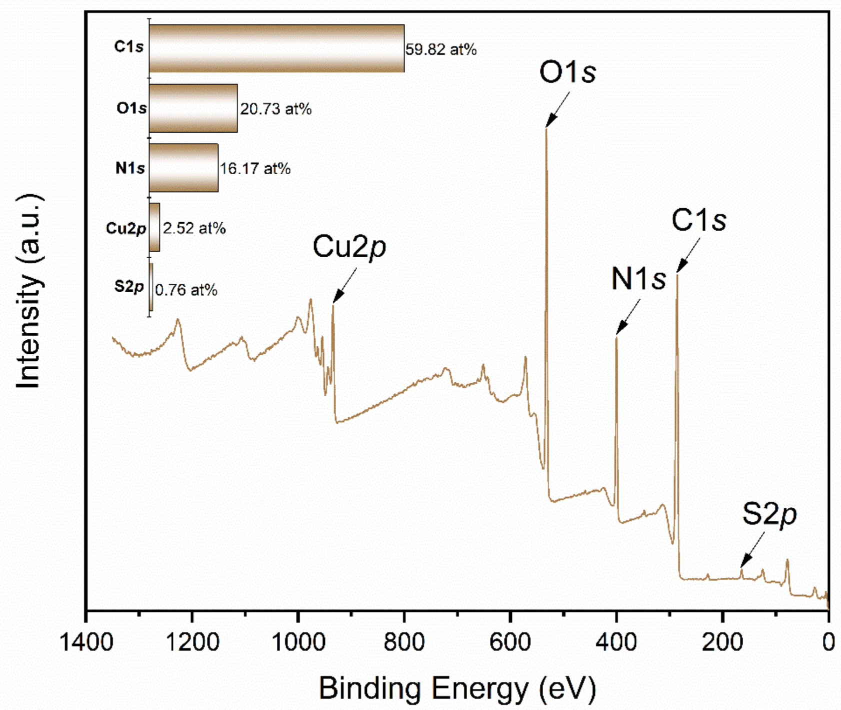

Figure S9. Elemental composition of CuP@CD revealed by X-ray photoelectron spectroscopy. XPS survey spectrum of CuP@CD showing the presence of C, N, O, S, and Cu elements. The corresponding binding-energy peaks verify the successful incorporation of Cu at porphyrinic sites within the carbon-dot framework.

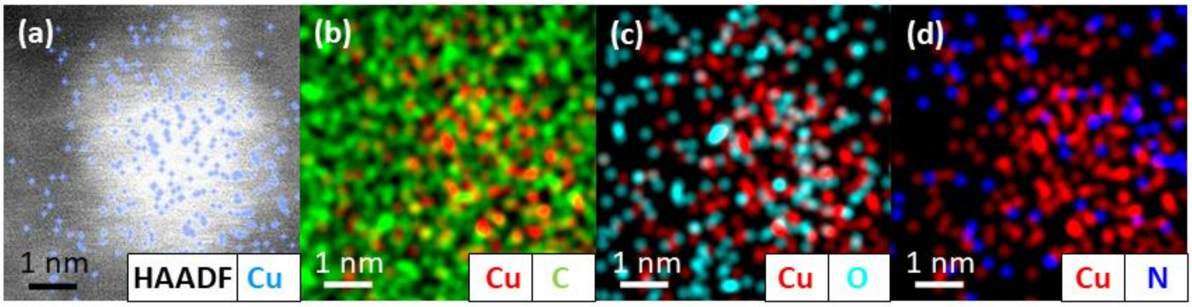

Figure S10. Elemental mapping of CuP@CD confirming homogeneous Cu distribution. High-resolution EDS elemental maps of CuP@CD showing the spatial distribution of **(a)** Cu overlaid on the HAADF image, **(b)** Cu and C, **(c)** Cu and O, and **(d)** Cu and N. The maps reveal a uniform dispersion of Cu atoms within the carbon-dot matrix and their close spatial association with surrounding heteroatoms, confirming the homogeneous incorporation of Cu in the nanoscale framework.

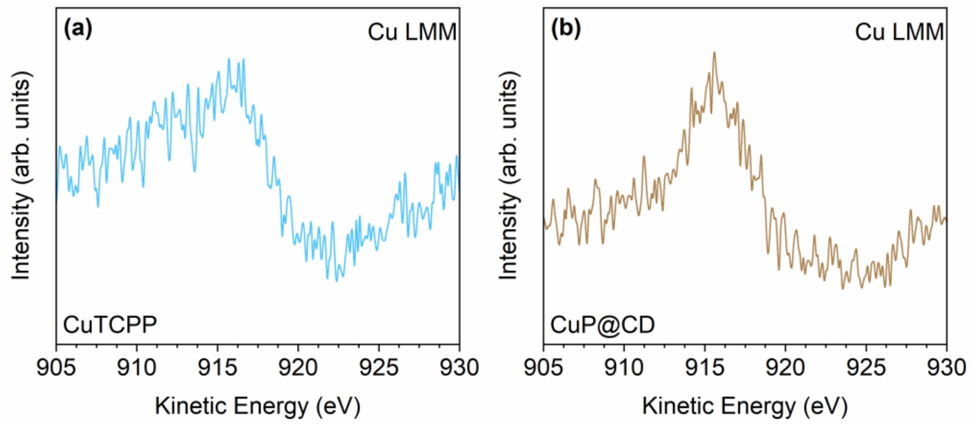

Figure S11. Auger electron spectroscopy confirming the +2 oxidation state of Cu in CuP@CD. Cu LMM Auger spectra obtained from XPS analysis for **(a)** CuTCPP reference and **(b)** CuP@CD. Similar peak positions confirm that Cu in CuP@CD is present predominantly in the Cu^{2+} oxidation state, as it is in the CuTCPP reference.

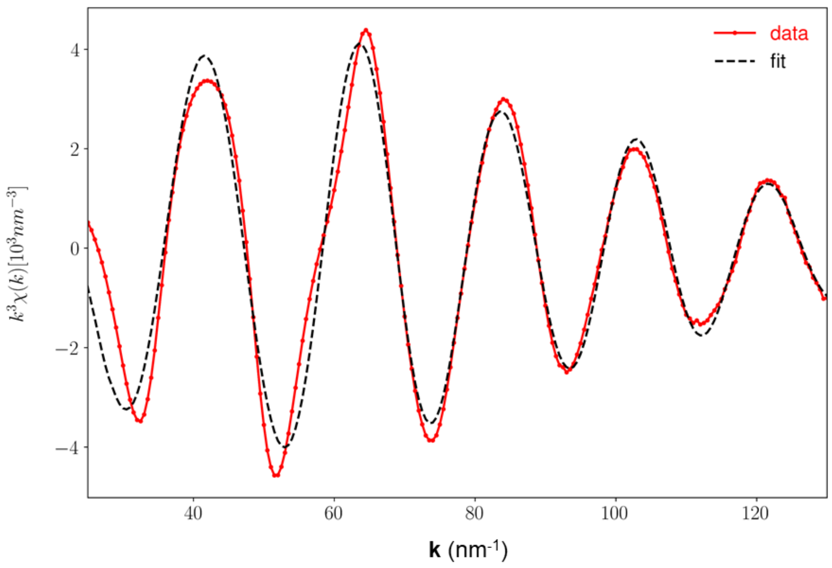

Figure S12. EXAFS fitting of CuP@CD confirming local Cu–N coordination. EXAFS of CuP@CD (red solid line) and its simulation (black dashed line). The fitting was performed using a single-shell Cu–N coordination model as described in the main text and Methods. The good agreement between the experimental and simulated data supports the presence of a well-defined coordination environment around the Cu centers, assigned to P_{680} sites at CD.

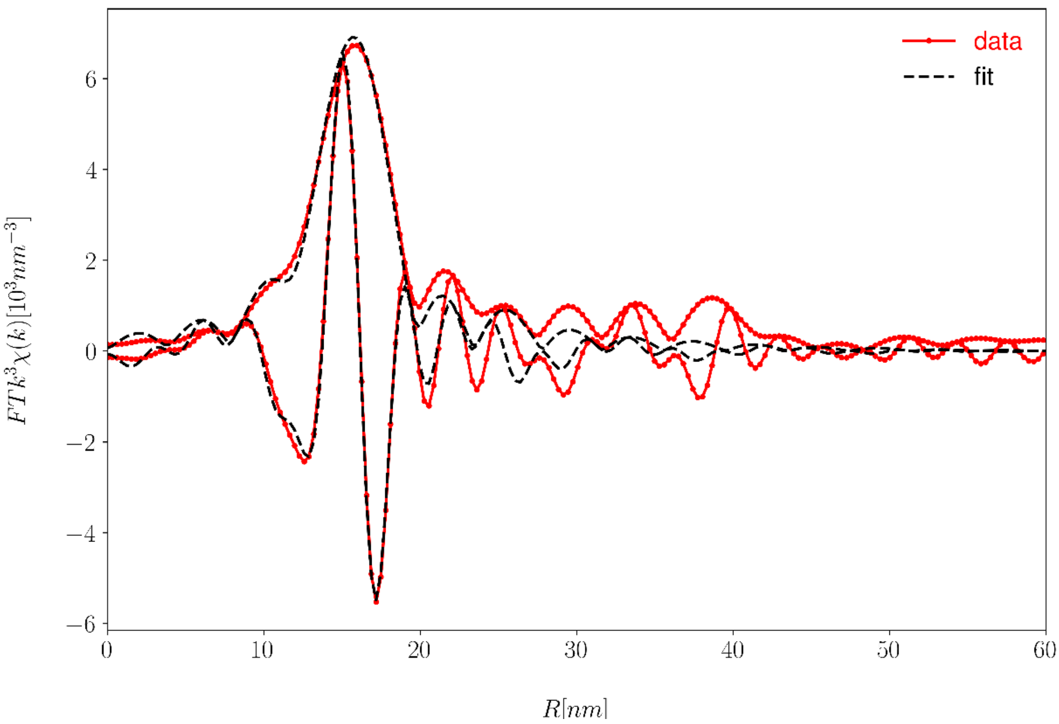

Figure S13. Fourier-transformed EXAFS analysis of CuTCPP confirming Cu–N coordination geometry. Fourier-transformed EXAFS data of CuTCPP (red solid line) and its simulation (black dashed line). The fitting was carried out using a single-shell Cu–N coordination model, consistent with the porphyrin structure of CuTCPP. The close agreement between experiment and simulation confirms the expected coordination geometry around the Cu center in the reference compound.

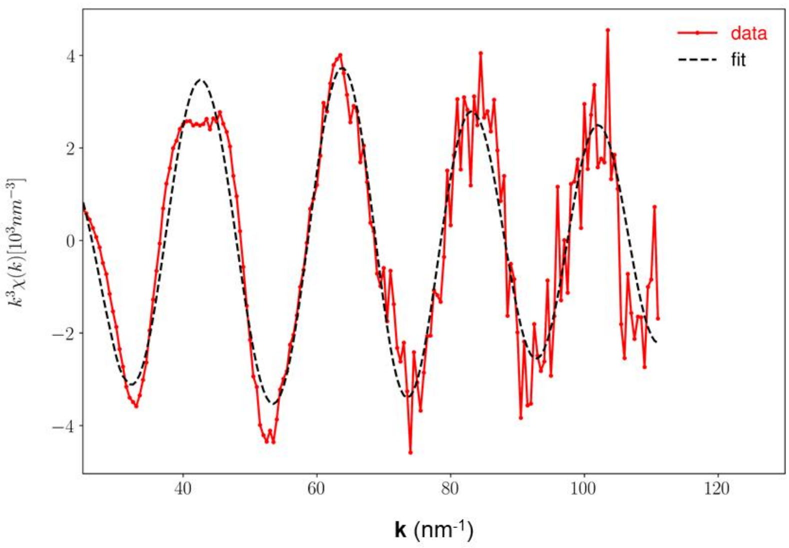

Figure S14. EXAFS fitting of the CuTCPP reference validating the Cu–N coordination model. EXAFS of CuTCPP (red solid line and its simulation (black dashed line). The fit reproduces the experimental oscillations well, confirming the structural reliability of the Cu–N coordination model used as a reference for analysis of CuP@CD.

Table S1. EXAFS fit results.

	**CuTCPP**	**CuP@CD**
N	3.4(4)	4.6(4)
R (nm)	0.199(1)	0.196(1)
σ^2 (10^{-5} nm^2)	3(1)	5.1(5)
S_0^2= 0.9		

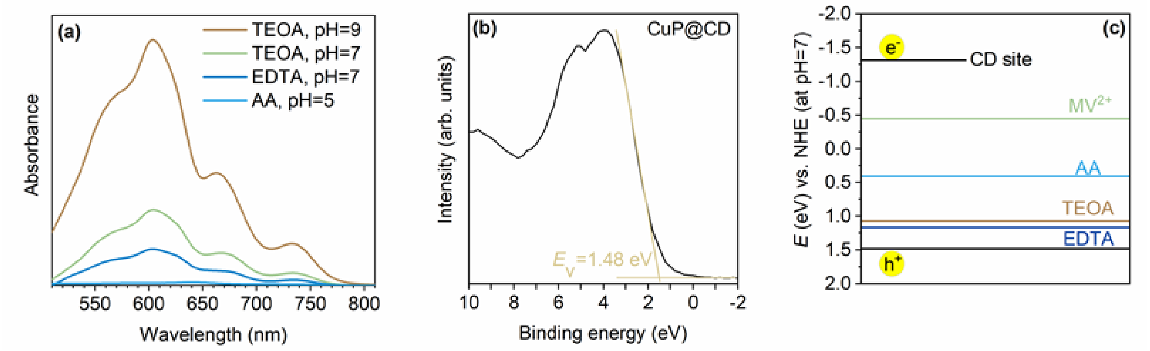

Figure S15. Exploration of the suitability of CuP@CD as a potential photocatalyst. (a) Generation of photo-reduced methyl viologen ($MV^{\bullet+}$) after photo-irradiation (100 mW/cm^2, solar light) using various sacrificial electron donors at different pH values. **(b)** Valence band XPS spectrum of CuP@CD. **(c)** Schematic illustration of the energy band structure of CuP@CD, shown together with the redox potentials of ascorbic acid (AA), ethylenediaminetetraacetic acid (EDTA), triethanolamine (TEOA), and methyl viologen (MV^{2+}).

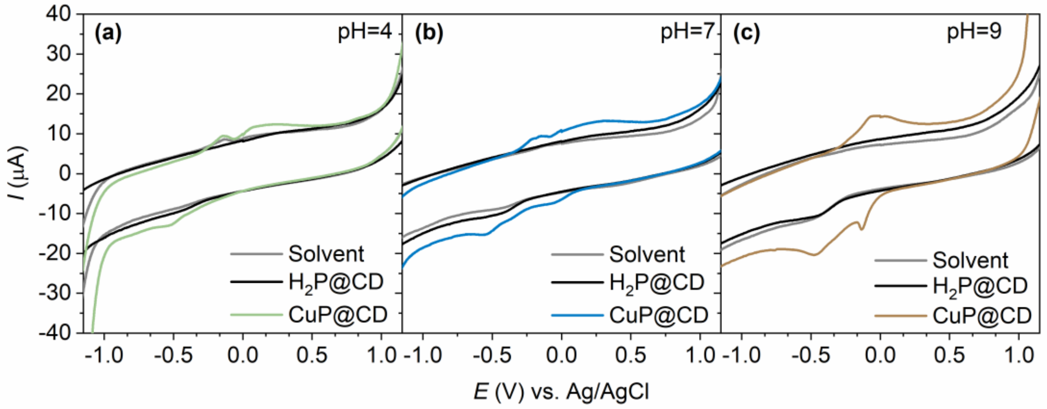

Figure S16. pH-dependent electrochemistry of CuP@CD and H_2P@CD. Cyclic voltammetry (100 mV s^{-1}) of solvent, H_2P@CD, and CuP@CD recorded at **(a)** pH 4, **(b)** pH 7, and **(c)** pH 9.

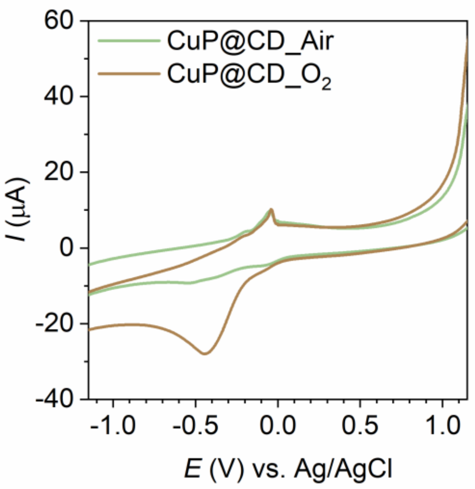

Figure S17. Electrochemical response of CuP@CD under air and O_2-saturated conditions. Cyclic voltammetry (100 mV s^{-1}) of CuP@CD in air- (green solid line) and in O_2-saturated (brown solid line) atmosphere (pH 9).

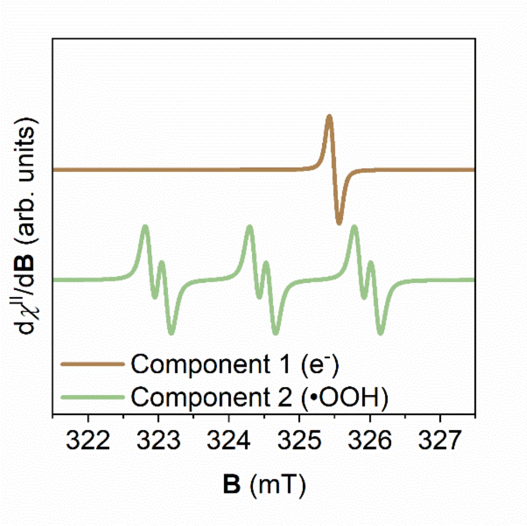

Figure S18. Simulated light-induced EPR components of CuP@CD under photocatalytic conditions. LEPR simulations of the various components of CuP@CD as observed in water (pH 9)/TEOA/PBN mixture.

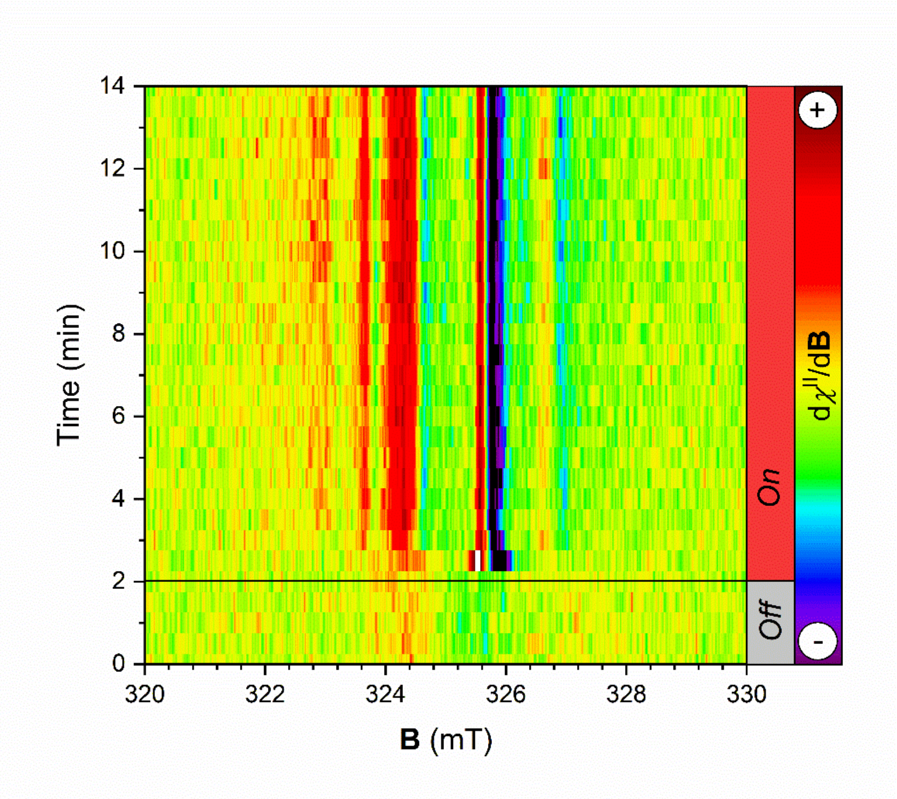

Figure S19. In situ LEPR monitoring of light-induced spin dynamics in H_2P@CD. The 2D color maps obtained from in situ LEPR experiment (CW X-band, 9.080 GHz, T = 293 K), showing the time evolution of the EPR signals when a dynamic light excitation sequence (off-on) is applied to a frozen solution of H_2P@CD in (pH 9)/TEOA/PBN mixture. The following experimental conditions were used during measurements: sample kept under dark conditions followed by in situ UV light exposure. The color coding indicates positive (+, red) to negative values (−, blue) of the EPR signal intensity ($d\chi^{II}/d\mathbf{B}$). The obtained LEPR signal does not exhibit hyperfine coupling constants characteristic of either $^{\bullet}$OOH or $^{\bullet}$OH radicals. Instead, it likely arises from the interaction of PBN with accumulated spin-active species on H_2P@CD.

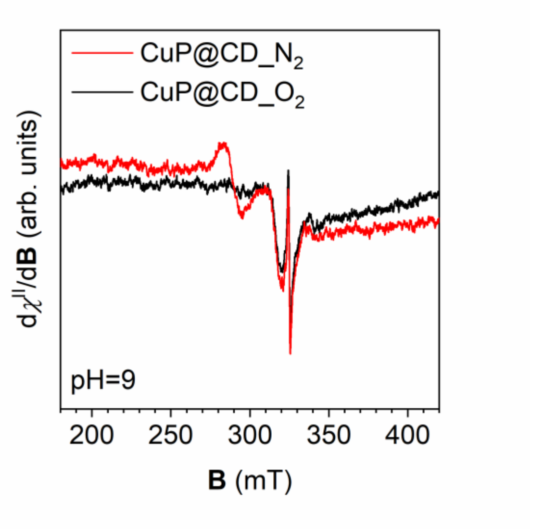

Figure S20. Low-temperature EPR spectra of CuP@CD under different atmospheres. EPR envelopes of CuP@CD under N_2- (red solid line) and O_2-saturated (black solid line) atmosphere (T = 80 K).

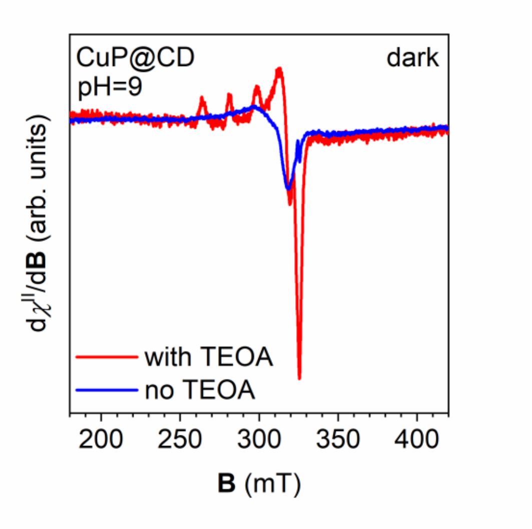

Figure S21. Effect of TEOA addition on the EPR signal of CuP@CD. EPR envelopes of CuP@CD before (blue solid line) and after (red solid line) addition of TEOA (T = 80K).

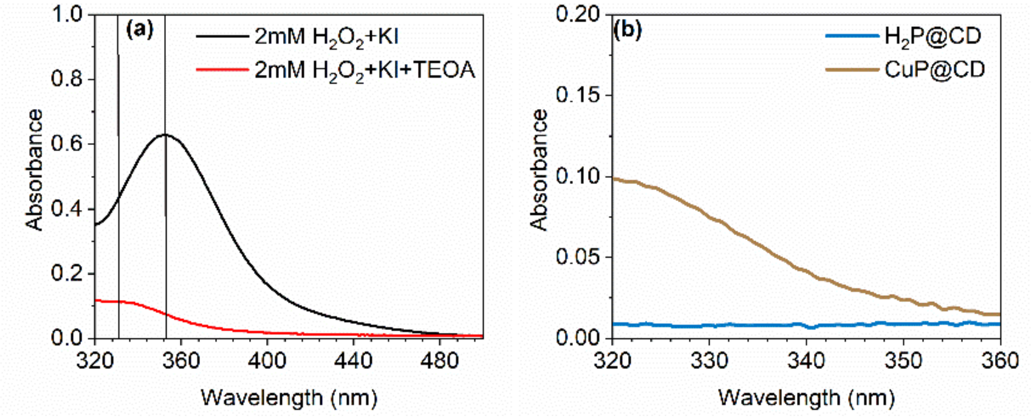

Figure S22. Optical detection of photocatalytically generated H_2O_2. (a) Absorption spectra of 2mM H_2O_2 and KI before and after addition of TEOA (pH 9). **(b)** Absorption spectra of H_2P@CD and CuP@CD after reaction (2 hours, solar simulator 100 mW cm^{-2}).

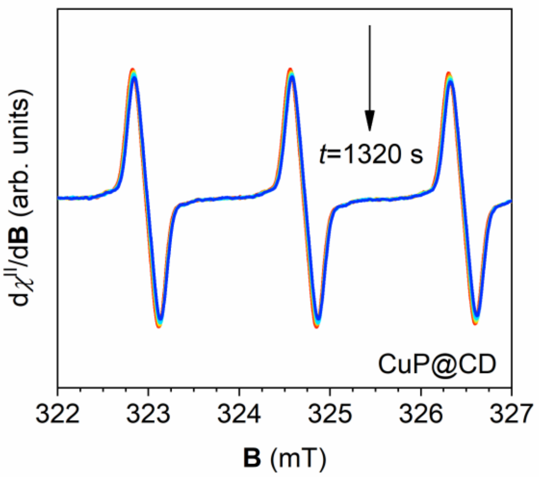

Figure S23. Time evolution of the TEMPO radical EPR signal in CuP@CD suspension in the dark. Evolution of the TEMPO radical EPR signal recorded for a water solution (pH 9) of CuP@CD containing TEOA (0.1 M) and TEMPO (1.95×10^{-4} M) at $T = 293$ K (dark).

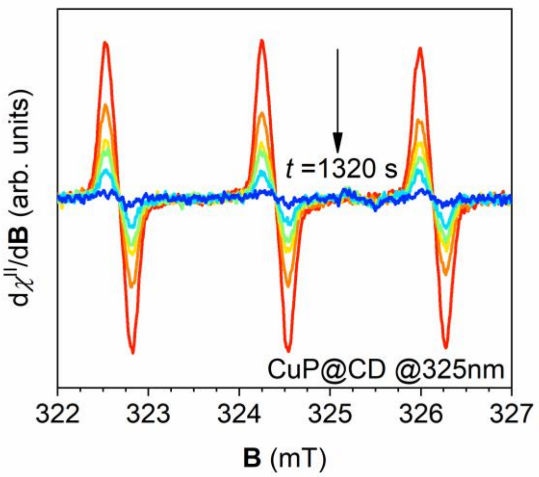

Figure S24. Time evolution of the TEMPO radical EPR signal in CuP@CD suspension under illumination. Evolution of the TEMPO radical LEPR signal recorded for a water solution (pH 9) of CuP@CD containing TEOA (0.1 M) and TEMPO (1.95×10^{-4} M) at $T = 293$ K (@325 nm, 40 mW).

H_2O_2 photoproduction calculation

Under photoexcitation, the O_2 molecules bound to the copper-containing active site (CuP) in the CuP@CD photocatalyst act as acceptors for the photogenerated e^- delivered from the carbon dot domain (CD). A series of O_2 reduction processes take place, which are highlighted by Eq. 1 to Eq. 4. Spin-active intermediates are formed during the O_2 reduction that can be trapped by spin-probes and analysed by EPR technique. We observed that the presence of $^{\bullet}OH$ radicals that may form from the photocatalytic H_2O_2 breakdown (see Eq. 5-6) under light irradiation (@325 nm) were excluded by both N-tert-Butyl-α-phenylnitrone (PBN) and 5,5-Dimethyl-1-pyrroline N-oxide (DMPO) spin-trap experiments. This evidence rules out a four-electron reduction pathway or a Fenton-like/electron-driven H_2O_2 decomposition. Therefore, formation of H_2O_2 as final product of the ORR catalyzed by CuP@CD occurs through one electron reduction pathway of O_2, which produces superoxide radical anions (Eq. 1, Eq. 2 and Eq. 3). These represent key steps in the ORR.[1]

$$h\nu(e^-) + O_2 \rightarrow O_2^{\bullet-} \qquad \text{Eq. 1}$$

$$H^+ + O_2^{\bullet-} \leftrightarrow {}^{\bullet}OOH \qquad \text{Eq. 2}$$

$$^{\bullet}OOH + {}^{\bullet}OOH \rightarrow O_2 + H_2O_2 \qquad \text{Eq. 3}$$

$$O_2^{\bullet-} + e^- + 2H^+ \rightarrow H_2O_2 \qquad \text{Eq. 4}$$

$$H_2O_2 + e^- \rightarrow {}^{\bullet}OH + OH^- \qquad \text{Eq. 5}$$

$$H_2O_2 + h\nu \rightarrow 2\ {}^{\bullet}OH \qquad \text{Eq. 6}$$

To elucidate the evolution of superoxide radicals in solution, we employed the TEMPO radical ($^{\bullet}T$) as a spin-probe and monitored the process of its oxidation by electron paramagnetic resonance (EPR) spectroscopy. The concentration of the superoxide intermediate species serves as an indicator of peroxide formation. The temporal decay of the TEMPO radical was recorded under 325 nm irradiation in an oxygen-saturated aqueous solution containing a hole scavenger and the photocatalyst. In the oxygen reduction reaction (ORR) pathway, the superoxide radical anion ($O_2^{\bullet-}$) generated *via* CuP@CD photocatalysis functions as a one-electron oxidant toward TEMPO, oxidizing TEMPO ($^{\bullet}N–O^{\bullet}$) to the corresponding oxoammonium cation ($TEMPO^+$, $=N^+–O^-$).

$$\text{TEMPO } ({}^{\bullet}N{-}O^{\bullet}) + O_2^{\bullet-} \rightarrow \text{TEMPO}^+ \ (=N^+{-}O^-) + O_2^{2-} \text{ (peroxide)}$$

The decay of the TEMPO signal at a fixed initial concentration (0.195 mM) was quantified by double integration of the EPR spectra, with intensities normalized to the square root of the applied microwave power (P). Measurements were first recorded under dark conditions (t = 0 s) and subsequently during irradiation as a function of time. At each time point, the integrated signal intensity (∬EPR Int./$\sqrt{P}$) was converted to TEMPO concentration using a pre-established calibration curve (presented in the following section). The decay profile exhibited second-order kinetic in TEMPO ([$^{\bullet}$T]), rather than first-order behaviour. This result is consistent with a mechanism in which O_2 activation proceeds *via* binding to the CuP active center. However, both TEMPO concentration and superoxide radical anion concentration are variables that dictates how fast the TEMPO disappearance occurs in solution. In support of this observation, purging the aqueous solution with N_2, thereby reducing considerably the dissolved O_2 concentration, led to a decrease in the TEMPO decay rate (see Figure 4f main manuscript text).

$$\text{TEMPO disappearance rate} = k_{obs}\,[\cdot T]^2\,[O_2^{\bullet-}]$$

Under steady-state conditions for the superoxide radical anion and with the TEMPO + $O_2^{\bullet-}$ step as rate-limiting, the reaction follows a pseudo–second-order rate law in TEMPO: $-d[{}^{\bullet}T]/dt = k\,[{}^{\bullet}T]^2$, being $k = k_{obs}\,[O_2^{\bullet-}]$ = const:

$$\frac{1}{[T^{\bullet}]_t} - \frac{1}{[T^{\bullet}]_0} = k \cdot t$$

Where $[^{\bullet}T]_t$ is the concentration of TEMPO radical at the time *t* under @325 nm irradiation, $[^{\bullet}T]_0$ is the concentration of TEMPO (fixed, 0.195 mM = 1.95×10^{-4} M) recorded initially (t = 0) under dark conditions and *k* is the second order rate constant ($M^{-1}s^{-1}$). By plotting $[^{\bullet}T]_t$ *vs* *t* (s) the linear fitting gives the *k* value, as angular coefficient (see Figure 4f, main manuscript text). Thus:

Rate constant $k = 48.56\ M^{-1}\ s^{-1}$

We can then calculate the amount of TEMPO radical left at the time *t*, being *t* = 3600 s (@325 nm)

$$[T^{\bullet}] = \frac{1}{\frac{1}{T_0}+kt} = \frac{1}{179944.2} = 5.55728\ \times 10^{-6}\ \mathrm{M} \qquad \text{Eq. 7}$$

The TEMPO consumed in 1 h = $[^{\bullet}T]_0 - [^{\bullet}T]_t = 1.95 \times 10^{-4} - 5.6 \times 10^{-6} = 1.894 \times 10^{-4}$ M, translates with the TEMPO radical that reacted (forming oxoammonium cation) with the photogenerated superoxide radicals at CuP site (Eq. 1-2 right) 1.894×10^{-4} M of $^{\bullet}OOH/O_2^{\bullet -}$ = 0.0001894 mmol/mL.

The amount of photoproduced H_2O_2 is finally calculated as follows:

<u>Assuming indirect two-steps, single-electron reduction ORR pathway (see Eq. 1 and Eq. 4):</u>

$^{\bullet}OOH/O_2^{\bullet -}$ = (0.0001894 mmol/mL h) · 0.120 mL = 0.0000227331 mmol/h

Amount of catalyst = 0.8 mg/mL taken 0.03 mL. Mass catalyst = 0.024 mg = 0.000024 g

(0.0000227331 mmol/h) / 0.000024 g = 0.94721 mmol/g h

Divided by the reaction volume (0.94721 mmol/g h) / (0.120 ml) = **7.89 mmol g^{-1} mL^{-1} h^{-1}**

<u>Assuming parallel radical recombination pathway (see Eq. 3):</u>

$$2^{\bullet}OOH \rightarrow H_2O_2 + O_2$$

½ $^{\bullet}OOH$ = (0.0000947 mmol/mL h) · 0.120 mL = 0.000011364 mmol/h

Amount of catalyst = 0.8 mg/mL taken 0.03 mL. Mass catalyst = 0.024 mg = 0.000024 g

(0.000011364 mmol/h) / 0.000024 g = 0.4735 mmol/g h

Divided by volume (0.94721 mmol/g h) / (0.120 ml) = **3.95 mmol g^{-1} mL^{-1} h^{-1}**

Conversion rate of H_2O_2 produced ORR

For the dissolved oxygen content, the solubility of O_2 in water at 25 °C is reported as 8.5 mg/l. Converting this to molar concentration:

$[O_2]$ = (8.5 mg/l) / (32 g/mol) = 0.266 mmol/l

In 120 µl: 0.266 mmol/l · 0.00012 l = 31.9 nmol

Therefore, the O_2-to-H_2O_2 conversion efficiency is:

Indirect two-step, single-electron reduction ORR pathway:
Conversion = (22.7 nmol / 31.9 nmol) · 100 = **71.2%**

Parallel radical recombination pathway:

Conversion = (11.35 nmol / 31.9 nmol) · 100 = **35.6%**

Apparent Quantum Yield (AQY) calculation

AQY was calculated following the methodology described in reference,[2] specifically using the number of photo-induced electrons involved in H_2O_2 formation relative to the total number of incident photons. Please note that the AQY value is reported without a standard deviation, as it was determined from a single experiment.

Irradiated area: $A = \pi r^2 = \pi \cdot (0.05)^2 = 0.007854\ \text{cm}^2$

Laser power: $P = 40\ \text{mW/cm}^2 \cdot 0.007854\ \text{cm}^2 = 0.31416\ \text{mW} = 0.00031416\ \text{J/s}$

Photon energy at 325 nm: $E_{\text{photon}} = (6.626 \times 10^{-34} \cdot 3 \times 10^{8}) / 325 \times 10^{-9} = 6.12 \times 10^{-19}\ \text{J}$

Total photons: $N_{\text{photons}} = (1.131\text{J}) / (6.12 \times 10^{-19}\ \text{J/photon}) = 1.85 \times 10^{18}$

Number of e^- involved in reaction: 0.00002273 mmol H_2O_2 / h = 2.273×10^{-8} mol

Since 2 e^- per H_2O_2, → total electrons = 4.546×10^{-8} mol

$N_e = 4.546 \times 10^{-8} \cdot 6.022 \times 10^{23} = 2.74 \times 10^{16}$ electrons

AQY = $(1.85 \times 10^{18} / 2.74 \times 10^{16}) \cdot 100$ = **1.48%**

Experimental procedure for generating the X-band EPR signal intensity calibration curve using the TEMPO radical as a spin concentration standard

A total of 9.9 mg of the TEMPO radical (2,2,6,6-Tetramethylpiperidine 1-oxyl, $C_9H_{18}NO$, 98% purity, CAS No. 2564-83-2; Sigma-Aldrich) was thoroughly dispersed in 10 mL of deionized water (CAS No. 7732-18-5; Sigma-Aldrich, Ultrapur) to prepare a stock solution with high TEMPO concentration ([TEMPO] = 6.34 mM). This stock solution was stored frozen (-18 °C) in dark, where it remained stable for months without radical degradation. A series of freshly prepared TEMPO solutions with varying concentrations were then obtained by serial dilution with deionized water of the above stock solution (*vide infra*). These dilutions were used to generate a calibration curve that correlate the EPR signal intensities with diverse TEMPO concentrations (from 0.0024 mM to 0.300 mM) and to define the EPR signal-detection range under specific sets of acquisition parameters used throughout the CuP@CD study.

Table S2. From the highly concentrated stock solution of TEMPO in water (6.34 mM), 0.1 mL were taken and diluted to 1.0 mL (final Volume) by adding deionized water (added 0.9 mL of H_2O). This solution is coded in the Table as Solution (**A**) = 0.634 mM

**Target [TEMPO] (mM)**	**Stock Volume (V_1, µL) Solution A**	**Water Volume (µL)**	**Notes**
**0.3000**	473.2	526.8	≈ **473 µL stock A + 527 µL water**
**0.2550**	402.2	597.8	≈ **402 µL stock A + 598 µL water**
**0.1940**	306.0	694.0	**306 µL stock A + 694 µL water**
**0.1500**	236.6	763.4	**≈ 237 µL stock A + 763 µL water**
**0.0970**	153.0	847.0	153 µL stock A + 847 µL water (**Solution B**)
**0.0485**	500.0 (from **Solution B**)	500.0	500 µL stock B + 500 µL water (**Solution C**)
**0.0243**	501.0 (from **Solution C**)	499.0	501 µL stock C + 499 µL water (**Solution D**)
**0.0024**	98.8 (from **Solution D**)	901.2	≈ 100 µL stock D + 900 µL water

Due to the high dielectric constant of water (ε = 78.4 at 298 K), which causes substantial microwave absorption, poor resonator coupling, and reduced sensitivity, particularly at room temperature, coupled with the water shallow microwave penetration depth, each TEMPO solution was loaded into WHEATON® capillary tubes (CAS number: 851321, 1-5 µL; Sigma-Aldrich). The capillaries featured a dark marked-scale grading, to ease consistency of the sample loading throughout the series. The loaded capillaries were finally inserted into 0.5 OD Wilmad® Suprasil EPR tubes (730-SQ-250M), which were then sealed using Precision Seal® rubber septa. In this way, the inner EPR tube atmosphere can further be controlled when needed, such as by saturating the local environment with N_2. Figure S24 shows, practically, the experimental set-up. X-band EPR measurements were all conducted at T = 293 K, using identical acquisition parameters across the TEMPO samples used in the calibration-curve settings: 100 kHz modulation frequency, 30 ms time constant, 8 × 100 gain, 0.3 mT modulation width, 0.500 mW microwave power, one scan and 1 minute of signal-acquisition time. The quality factor (Q) of the X-band EPR resonator cavity remained within the range 6100 - 6500 across the tested solutions under X-band microwave irradiation (for the empty X-band resonator cavity $Q \cong 8800$) and optimal loading of 1 µL. In fact, for obtaining good signal-intensity vs concentration calibration curve, all these experimental variables must be optimized, because the detected EPR signal voltage (V) depends on:

$$V_{EPR} = K \cdot \sqrt{P_0} \cdot Q \cdot \eta \cdot \chi'' \cdot G \qquad \text{(Eq. 8)}$$

It is convenient to divide the EPR signal voltage (V), namely the EPR intensity, for the square root of the applied microwave power (expressed in microwatt, µW) such that:

$$V_{EPR} / \sqrt{P_0} = K \cdot Q \cdot \eta \cdot \chi'' \cdot G \qquad \text{(Eq. 9)}$$

K = detector sensitivity, $\sqrt{P_0}$ = square root of applied microwave power (must be far from saturating conditions), Q = cavity quality factor, η = filling factor (dimensionless, represents the fraction of microwave magnetic field interacting with the sample), χ'' = imaginary part of the RF susceptibility, G = gain of the detection system. The resulting EPR spectra ($d\chi^{II}/d\mathbf{B}$, derivative of the magnetic susceptibility with respect to field) obtained by varying TEMPO concentration are shown in Figure S25. These resonance signals were divided for the square root of microwave power, as shown in Eq. 9, and then double integrated (D.I.). The resulting values (D.I.) were subsequently plotted against the known TEMPO concentrations (mM) to generate the linear calibration curve (y (D.I.) = a + b*x (mM), with coefficients a = 16.2 and b = 11963.1) shown Figure S26.

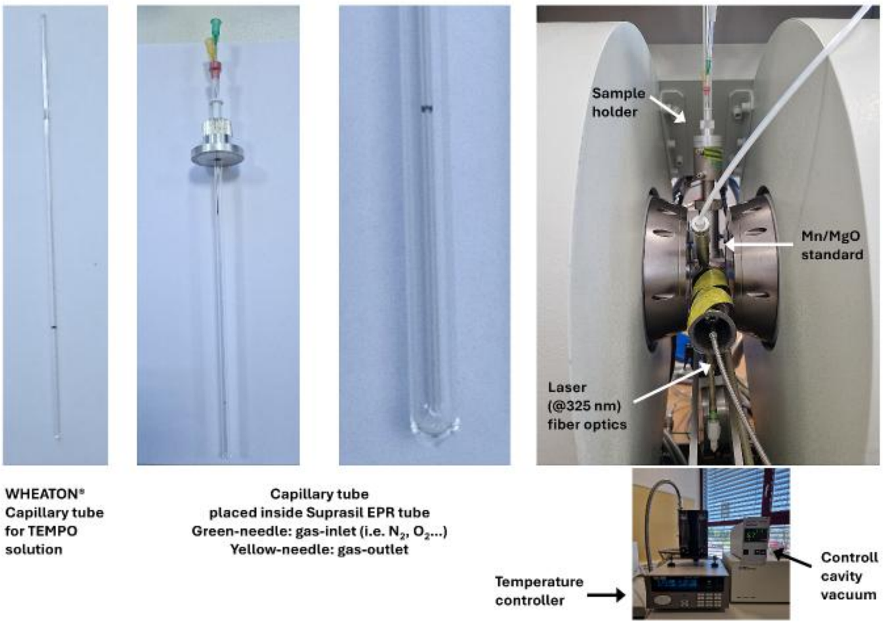

Figure S25. Experimental setup for EPR-based TEMPO radical measurements. Experimental set-up for the TEMPO radical measurements performed with X-band EPR spectroscopy as used throughout the study, including the determination of the TEMPO EPR signal calibration curve.

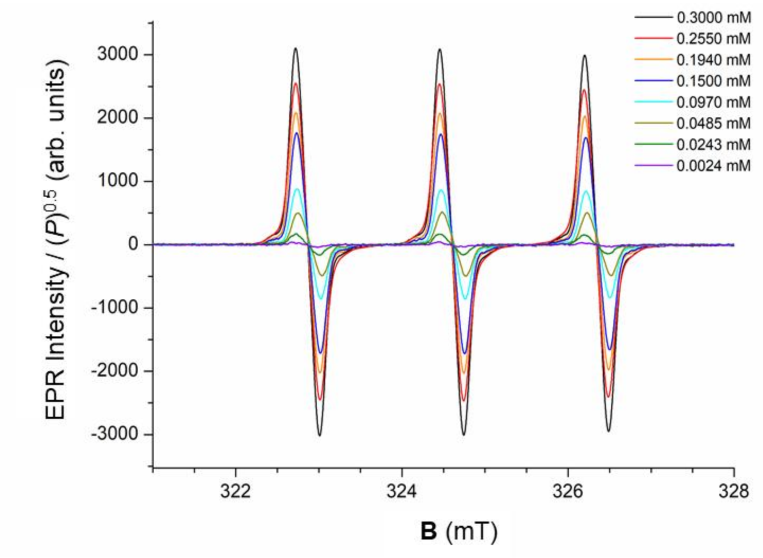

Figure S26. Calibration measurements of the TEMPO radical EPR signal intensity. X-band (9.08 GHz) TEMPO radical EPR signal recorded for various TEMPO concentrations.

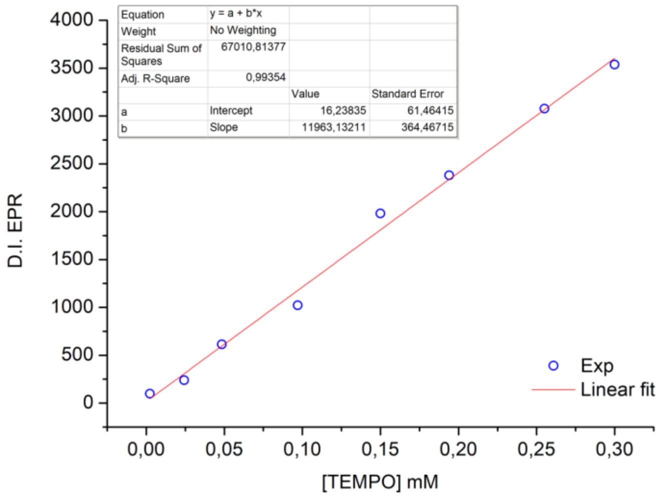

Figure S27. Calibration curve of TEMPO concentration versus EPR signal intensity. Linear plot of the double-integrated EPR intensities (open circles) of the TEMPO resonance signals *vs.* concentration of TEMPO (mM). The red line shows the linear fitting based on coefficient values (a, b) given in the plot.

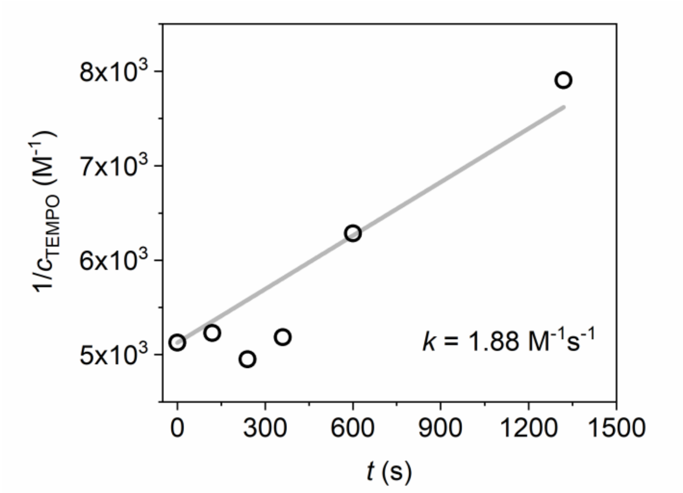

Figure S28. Kinetic analysis of TEMPO signal decay under photocatalytic conditions. Second-order kinetic analysis of the TEMPO signal decay in a water solution (pH 9) containing TEOA (0.1 M) and TEMPO (1.95×10^{-4} M) at $T = 293$ K (@325 nm, 40 mW).

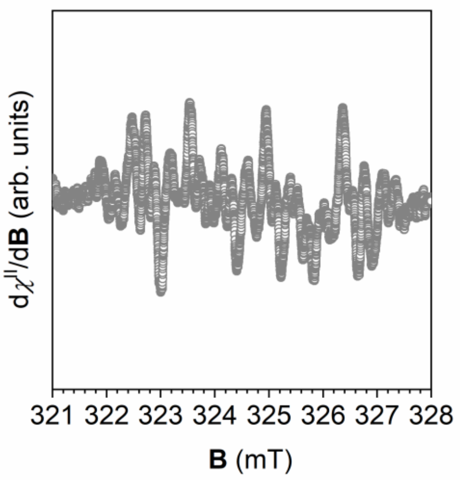

Figure S29. Light-induced EPR spectrum of decomposed CuP@CD under photocatalytic conditions. LEPR signal of decomposed CuP@CD in water (pH 9) containing TEMPO (1.95×10^{-4} M) at $T = 293$ K (@325 nm, 40 mW, irradiation time $t = 1320$ s).

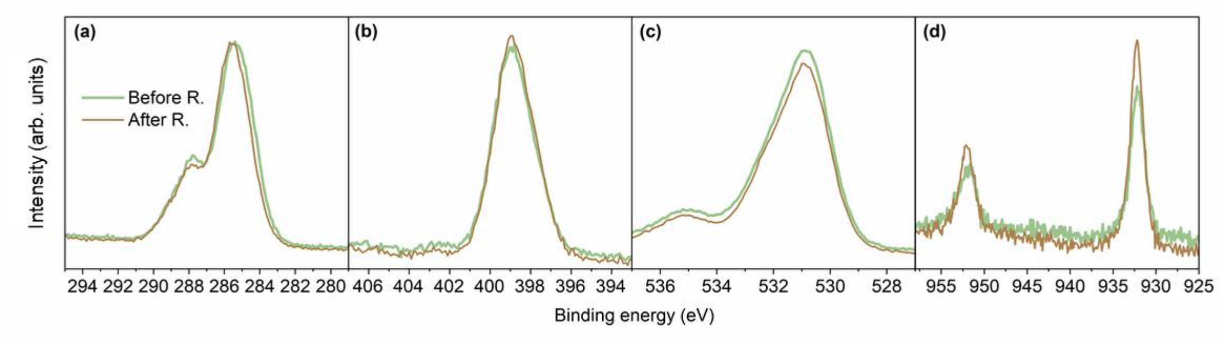

Figure S30. Comparison of high-resolution XPS spectra of CuP@CD before and after photocatalytic reaction. High-resolution XPS spectra of CuP@CD before and after the reaction: **(a)** C 1*s*, **(b)** N 1*s*, **(c)** O 1*s*, and **(d)** Cu 2*p*.

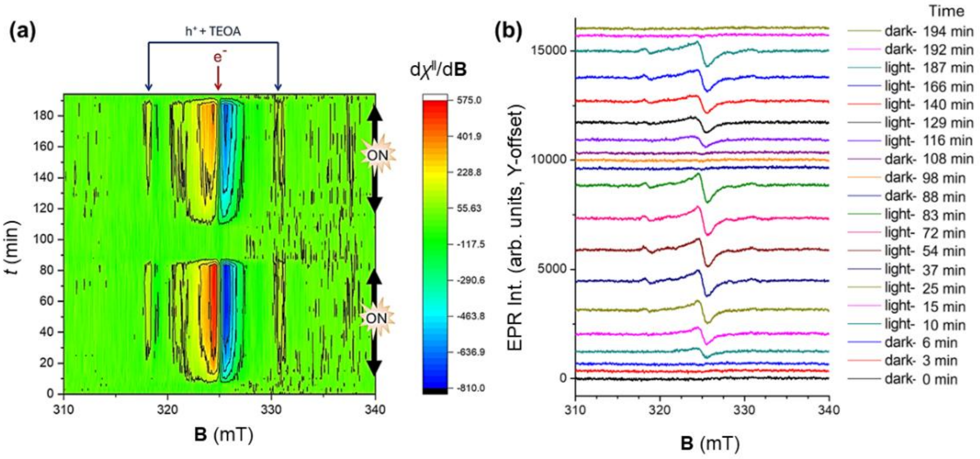

Figure S31. In situ LEPR monitoring of light-induced charge carrier dynamics in CuP@CD. (a) 2D heat map generated from X-band (9.0795 GHz) measurements of CuP@CD in 0.1 M TEOA in water at pH 9 and at 80 K, under light on (λ_{ex} = 325 nm) and light off conditions. The 2D plot shows formation of photoexcited charges (electrons/holes) and their disappearance when light is off to highlight the reversibility of the photoexcitation. **(b)** The individual EPR resonance signals recorded during the light off to light on (λ_{ex} = 325 nm) cycles from which the 2D heat map shown in panel **(a)** has been generated.

Table S3. Literature overview.

	Photocatalyst	Light source	Conditions	H_2O_2 (μmol/g·h)	Volume (ml)	H_2O_2 (μmol/g·ml·h)	Ref
1	PM-CDs-30 (10 mg)	34.8 mW/cm^2, λ≥420 nm	H_2O	1776	20	88.8	2
2	$CN_{1.8}$/ICT/CDs (8 mg)	λ≥420 nm	H_2O	2203	15	146.9	3
3	CD-AQ (- mg)	300W Xe lamp, AM 1.5G	H_2O (O_2)	1188	40	29.7	4
4	SnS_2/In_2S_3/CDs (20 mg)	λ≥420 nm	H_2O	1112	15	74.1	5
5	HTNT-CD (20 mg)	350 W Xe lamp (λ>420 nm)	H_2O/EtOH	3420	15	228.0	6
6	CQD-CTF (25 mg)	300 W Xe lamp (λ>420 nm)	H_2O (O_2)	1036	100	10.4	7
7	CDs/CMP (20 mg)	300 W Xe lamp	H_2O/FFA (O_2)	8543	50	170.9	8
8	Au/TiO_2 (200 mg)	λ>300 nm	H_2O/EtOH	2080	200	10.4	9
9	COF-TAPB-BPDA (5 mg)	300W Xe lamp, λ>420 nm	H_2O/BA (O_2)	1240	25	49.6	10
10	Py-Py-COF (5 mg)	300W Xe lamp, λ>420 nm	H_2O/BA (O_2)	1242	5	248.4	11
11	TiO_2/PEI/AgNP (50 mg)	300W Xe lamp, λ>420 nm	H_2O	1605	50	32.1	12
12	CTF-NS-5BT (20 mg)	300W Xe lamp, λ>420 nm	H_2O/BA (O_2)	1630	20	81.5	13
13	NMT400 (20 mg)	300W Xe lamp, AM 1.5G	H_2O/EtOH (O_2)	1695	50	33.9	14
14	EBA-COF (10 mg)	50W LED lamp, λ~420 nm	H_2O/EtOH (O_2)	1830	2.5	732	15
15	ACN (25 mg)	300W Xe lamp, AM 1.5G	H_2O/IpOH (O_2)	1874	50	37.5	16
16	g-C_3N_4-N_3C (10 mg)	300W Xe lamp, AM 1.5G	H_2O/IpOH (O_2)	1915	100	19.2	17
17	CDs1-NCN (10 mg)	300W Xe lamp, 420 nm≤ λ ≤700 nm	H_2O	1938	15	129.2	18
18	ZnO/COF (- mg)	300W Xe lamp, AM 1.5G	H_2O/EtOH (O_2)	2443	-	-	19
19	Pd/A/$BiVO_4$ (100 mg)	300W Xe lamp, λ>420 nm	H_2O/PBS/EtOH (O_2)	2577	100	25.8	20
20	TiO_2/Bi_2O_3 (20 mg)	300W Xe lamp, 350 nm≤ λ ≤780 nm	H_2O/FAA (O_2)	2875	50	57.5	21
21	TPB-DMTP-COF (10 mg)	300W Xe lamp, λ>420 nm	H_2O (O_2)	2882	50	57.6	22
22	TiO_2/MoS_x-Au (10 mg)	300W Xe lamp	H_2O/EtOH (O_2)	30 440	100	304.4	23
23	CQDs-NH (10 mg)	300W Xe lamp, AM 1.5G	H_2O/EtOH	2974	50	59.5	24

24	$SrCoO_3$-MoS_2 (50 mg)	300W Xe lamp	H_2O/glycerol (O_2)	15900	50	318	[25]
25	N-ethyl-2-piperazinone (100 mg)	420 nm ≤ λ ≤ 700 nm	H_2O	2885	110	26.2	[26]
26	S-doped CDs (25 mg)	300W Xe lamp, AM 1.5G	H_2O	2060	40	51.5	[27]
27	COF/In_2S_3 (30 mg)	360 W Xe lamp	H_2O	5713	30	190.4	[28]
28	Conjugated polymers (1 mg)	300W Xe lamp, λ>400 nm	H_2O	1365	50	27.3	[29]
29	COFs (TT-COF-X (5 mg)	300W Xe lamp, λ>400 nm	H_2O (O_2)	3406	50	68.1	[30]
30	PD-COF2 (- mg)	300W Xe lamp	H_2O (O_2)	6103	8	762.9	[31]
31	carbon/potassium poly(heptazine imide) – Pt (5 mg)	50 W, λ = 410 nm	3.5% w/w glycerine in H_2O	3940	2	1970	[32]
32	C_3N_5 (20 mg)	300W Xe lamp, λ>420 nm	H_2O/EtOH	3810	40	95.2	[33]
33	RF523 (50 mg)	300W Xe lamp, AM 1.5G	Water	50	30	1.7	[34]
34	O/K-CN (25 mg)	300W Xe lamp, λ>420 nm	H_2O/IpOH	15470	50	309.4	[35]
35	Sb-SAPC (- mg)	300W Xe lamp, λ>420 nm	Water	588	50	11.8	[36]
	**CuP@CD (0.024)**	**325 nm 40mW/cm^2**	**H_2O/TEOA pH 9**	**947**	**0.120**	**7893.5**	**-**

Reference:

1 Wang, Y. *et al.* Si/Carbon-dots with Surface N-C Sites Promoting Proton and Electron Transfers in Oxygen Reduction Reaction. *Angew. Chem.-Int. Edit.*, e202509790, doi:10.1002/anie.202509790 (2025).

2 Wu, Q. *et al.* A metal-free photocatalyst for highly efficient hydrogen peroxide photoproduction in real seawater. *Nat. Commun.* **12**, 483, doi:10.1038/s41467-020-20823-8 (2021).

3 Li, Y. *et al.* Photo-charge regulation of metal-free photocatalyst by carbon dots for efficient and stable hydrogen peroxide production. *J. Mater. Chem. A* **9**, 25453, doi:10.1039/d1ta07802j (2021).

4 Gu, M. *et al.* Solar-to-hydrogen peroxide conversion of photocatalytic carbon dots with anthraquinone: Unveiling the dual role of surface functionalities. *Appl. Catal. B-Environ.* **312**, doi:10.1016/j.apcatb.2022.121379 (2022).

5 Li, Y. *et al.* Interface photo-charge kinetics regulation by carbon dots for efficient hydrogen peroxide production. *J. Mater. Chem. A* **9**, 515, doi:10.1039/d0ta10231h (2021).

6 Ma, R. *et al.* Solid acids accelerate the photocatalytic hydrogen peroxide synthesis over a hybrid catalyst of titania nanotube with carbon dot. *Appl. Catal. B-Environ.* **244**, 594, doi:10.1016/j.apcatb.2018.11.087 (2019).

7 Yang, Y. *et al.* Carbon Quantum Dots Confined into Covalent Triazine Frameworks for Efficient Overall Photocatalytic H_2O_2 Production. *Adv. Funct. Mater.* **34**, doi:10.1002/adfm.202400612 (2024).

8 Li, X. *et al.* Self-Floating Photocatalytic System for Highly Efficient Hydrogen Peroxide Production and Organic Synthesis on Carbon Dots Decorated Conjugated Microporous Polymer. *Adv. Funct. Mater.* **34**, doi:10.1002/adfm.202316773 (2024).

9 Teranishi, M., Naya, S. & Tada, H. In situ liquid phase synthesis of hydrogen peroxide from molecular oxygen using gold nanoparticle-loaded titanium(IV) dioxide photocatalyst. *J. Am. Chem. Soc.* **132**, 7850, doi:10.1021/ja102651g (2010).

10 Yang, T., Chen, Y., Wang, Y., Peng, X. & Kong, A. Weakly Hydrophilic Imine-Linked Covalent Benzene-Acetylene Frameworks for Photocatalytic H_2O_2 Production in the Two-Phase System. *ACS Appl. Mater. Interfaces* **15**, 8066, doi:10.1021/acsami.2c20506 (2023).

11 Sun, J. *et al.* Pyrene-Based Covalent Organic Frameworks for Photocatalytic Hydrogen Peroxide Production. *Angew. Chem.-Int. Edit.* **62**, e202216719, doi:10.1002/anie.202216719 (2023).

12 Li, X. *et al.* Boosting photocatalytic H_2O_2 production in pure water over a plasmonic photocatalyst with polyethylenimine modification. *J. Mater. Chem. A* **11**, 1503, doi:10.1039/d2ta08203a (2023).

13 Yu, X. *et al.* Electronic Tuning of Covalent Triazine Framework Nanoshells for Highly Efficient Photocatalytic H_2O_2 Production. *Adv. Sus. Systems* **5**, 2100184, doi:10.1002/adsu.202100184 (2021).

14 Yang, C., Wan, S., Zhu, B., Yu, J. & Cao, S. Calcination-regulated Microstructures of Donor-Acceptor Polymers towards Enhanced and Stable Photocatalytic H_2O_2 Production in Pure Water. *Angew. Chem.-Int. Edit.* **61**, e202208438, doi:10.1002/anie.202208438 (2022).

15 Zhai, L. *et al.* Constructing Synergistic Triazine and Acetylene Cores in Fully Conjugated Covalent Organic Frameworks for Cascade Photocatalytic H_2O_2 Production. *Chem. Mater.* **34**, 5232, doi:10.1021/acs.chemmater.2c00910 (2022).

16 Zheng, Y. *et al.* Plasma-induced hierarchical amorphous carbon nitride nanostructure with two N_2 C-site vacancies for photocatalytic H_2O_2 production. *Appl. Catal. B-Environ.* **311**, 121372, doi:10.1016/j.apcatb.2022.121372 (2022).

17 Xue, Y. *et al.* Construction of g-C_3N_4 with three coordinated nitrogen (N_{3C}) vacancies for excellent photocatalytic activities of N_2 fixation and H_2O_2 production. *Chem. Eng. J.* **457**, doi:10.1016/j.cej.2022.141146 (2023).

18 Wei, K. *et al.* Carbon dots with different energy levels regulate the activity of metal-free catalyst for hydrogen peroxide photoproduction. *J. Colloid Interface Sci.* **616**, 769, doi:10.1016/j.jcis.2022.02.107 (2022).

19 Zhang, Y. *et al.* ZnO/COF S-scheme heterojunction for improved photocatalytic H_2O_2 production performance. *Chem. Eng. J.* **444**, 136584, doi:10.1016/j.cej.2022.136584 (2022).

20 Sun, M. *et al.* Bifunctional Pd-Ox Center at the Liquid–Solid–Gas Triphase Interface for H_2O_2 Photosynthesis. *ACS Catalysis* **12**, 2138, doi:10.1021/acscatal.1c05324 (2022).

21 He, B. *et al.* Cooperative Coupling of H_2O_2 Production and Organic Synthesis over a Floatable Polystyrene-Sphere-Supported TiO_2/Bi_2O_3 S-Scheme Photocatalyst. *Adv. Mater.* **34**, e2203225, doi:10.1002/adma.202203225 (2022).

22 Li, L., Xu, L., Hu, Z. & Yu, J. C. Enhanced Mass Transfer of Oxygen through a Gas–Liquid–Solid Interface for Photocatalytic Hydrogen Peroxide Production. *Adv. Funct. Mater.* **31**, 2106120, doi:10.1002/adfm.202106120 (2021).

23 Zhang, X. *et al.* Enhancing photocatalytic H_2O_2 production with Au co-catalysts through electronic structure modification. *Nat. Commun.* **15**, 3212, doi:10.1038/s41467-024-47624-7 (2024).

24 Han, W. *et al.* Surface engineered carbon quantum dots for efficient photocatalytic hydrogen peroxide production. *Appl. Catal. B-Environ.* **350**, doi:10.1016/j.apcatb.2024.123918 (2024).

25 He, Y. *et al.* Efficient photocatalytic H_2O_2 production and green oxidation of glycerol over a $SrCoO_3$-incorporated catalyst. *Appl. Catal. B-Environ.* **361**, 124565, doi:10.1016/j.apcatb.2024.124565 (2025).

26 He, T. *et al.* A metal-free cascaded process for efficient H_2O_2 photoproduction using conjugated carbonyl sites. *Nat. Commun.* **15**, 7833, doi:10.1038/s41467-024-52162-3 (2024).

27 Qin, H. *et al.* Boosted photocatalytic H_2O_2 production in pure water with amino-modified N, S-doped carbon dots. *Chem. Eng. J.* **499**, doi:10.1016/j.cej.2024.156239 (2024).

28 Qiu, J. *et al.* COF/In_2S_3 S-Scheme Photocatalyst with Enhanced Light Absorption and H_2O_2-Production Activity and fs-TA Investigation. *Adv. Mater.* **36**, e2400288, doi:10.1002/adma.202400288 (2024).

29 Yan, H. *et al.* Enhancing Photosynthesis Efficiency of Hydrogen Peroxide by Modulating Side Chains to Facilitate Water Oxidation at Low-Energy Barrier Sites. *Adv. Mater.* **36**, e2311535, doi:10.1002/adma.202311535 (2024).

30 Yao, Y. *et al.* Synergistic Tri-efficiency Enhancement Utilizing Functionalized Covalent Organic Frameworks for Photocatalytic H_2O_2 Production. *Small* **20**, e2404885, doi:10.1002/smll.202404885 (2024).

31 Yue, J. Y. *et al.* Phenanthridine-based Covalent Organic Frameworks for Boosting Overall Solar H_2O_2 Production. *Angew. Chem.-Int. Edit.*, e202417115, doi:10.1002/anie.202417115 (2024).

32 Pelicano, C. M. *et al.* Rational design of a carbon/potassium poly(heptazine imide) heterojunction for enhanced photocatalytic H_2 and H_2O_2 evolution. *J. Mater. Chem. A* **12**, 475, doi:10.1039/d3ta05701a (2024).

33 Li, Z. *et al.* Dipole field in nitrogen-enriched carbon nitride with external forces to boost the artificial photosynthesis of hydrogen peroxide. *Nat. Commun.* **14**, 5742, doi:10.1038/s41467-023-41522-0 (2023).

34 Shiraishi, Y. *et al.* Resorcinol-formaldehyde resins as metal-free semiconductor photocatalysts for solar-to-hydrogen peroxide energy conversion. *Nat. Mater.* **18**, 985, doi:10.1038/s41563-019-0398-0 (2019).

35 Liu, W. *et al.* Unraveling the Mechanism on Ultrahigh Efficiency Photocatalytic H_2O_2 Generation for Dual-Heteroatom Incorporated Polymeric Carbon Nitride. *Adv. Funct. Mater.* **32**, 2205119, doi:10.1002/adfm.202205119 (2022).

36 Teng, Z. *et al.* Atomically dispersed antimony on carbon nitride for the artificial photosynthesis of hydrogen peroxide. *Nat. Catal.* **4**, 374, doi:10.1038/s41929-021-00605-1 (2021).
