# Supplementary material for: Enzyme-inspired single-atom photocatalysis for oxygen reduction to hydrogen peroxide
Source: Nat Commun. 2025 Dec 7;16:10949. doi: 10.1038/s41467-025-67189-3 (PMC12686429; doi:10.1038/s41467-025-67189-3)
Supplement: Supplementary file 2 — Description of Additional Supplementary File [file 41467_2025_67189_MOESM2_ESM.pdf]

Description of Additional Supplementary file

Supplementary Data 1 – Fitting files for Supplementary Figures S12–S14

ZIP archive containing the raw and fitted datasets, associated command/fit files, and processing scripts used for analysis of the data shown in Supplementary Figures S12–S14. These files enable full reproducibility of the complex spectral analysis.
